# The Effects of the Situated Simulation Program on the Cultural Competence of Hemodialysis Nurses: A Quasi-Experimental Study

**DOI:** 10.3390/healthcare11192609

**Published:** 2023-09-22

**Authors:** Jui-Chin Hsu, Min-Shu Wang, Pao-Yu Wang, Shu-Yu Lian, Mei-Hsiang Lin

**Affiliations:** 1 Department of Nursing, Cheng Hsin General Hospital, Taipei City 112, Taiwan; 2 Department of Medical Education Clinical Skills Center, Mackay Memorial Hospital, New Taipei City 251, Taiwan; 3 Department of Nursing, MacKay Junior College of Medicine, Nursing and Management, New Taipei City 116, Taiwan; 4 School of Nursing, National Taipei University of Nursing and Health Sciences, Taipei City 112, Taiwan

**Keywords:** situated simulation, cultural competence, hemodialysis, nurses

## Abstract

The rise in the number of hemodialysis (HD) patients underscores the importance of culturally competent HD nurses. This study aimed to examine the effectiveness of a situated simulation program on HD nurses’ cultural competence. This was a quasi-experimental pilot study with a total of 40 participants who met the inclusion criteria from an HD center in northern Taiwan. Participants took part in two separate 3 h education programs. The first program focused on the basic concepts of cultural competence, while the second program involved situated simulations utilizing the Gather–Analyze–Summarize (GAS) method of debriefing. The generalized estimating equations (GEEs) were employed to estimate the intervention effect. The baseline scores were divided into low-score and high-score groups using the median score for subgroup analysis. The subgroup analysis revealed that a significant group-time interaction was identified regarding cultural competence and subscale, verifying the situated simulation’s immediate effect. In this study, an integration of the GAS method of debriefing and situated simulation teaching was implemented. The results showed that this approach empowered HD nurses with the ability to foster positive attitudes and demonstrate professional expertise in an organized manner when facing similar clinical scenarios in the future.

## 1. Introduction

End-stage renal disease (ESRD) represents the final manifestation of chronic kidney disease. Taiwan’s prevalence and incidence rates, as reported by the United States Renal Data System [1], rank highest globally. In terms of the prevalence of dialysis in Taiwan, the number of dialysis patients increased from 77,746 in 2015 to 86,840 in 2019 [2]. The rise in chronic kidney disease cases is a growing concern in various regions worldwide, leading to considerably high mortality rates and its emergence as a common public health issue [3]. Hemodialysis (HD) remains the most common treatment in renal replacement therapy, offering symptom relief and life-sustaining support. However, the dialysis population faces increased mortality risks due to the presence of multiple comorbidities [1]. In terms of the psychological adjustment process among HD patients, although hemodialysis is considered a common treatment method for HD patients, it is always accompanied by some complications. Physical, mental and social problems may increase in these patients during hemodialysis, and patients may face significant challenges and problems, including psychological problems [4]. Understanding the factors that influence the incidence and prevalence of psychological symptoms in patients with chronic disease is necessary [5]. The issue of psychological problems among HD patients has been debated in recent related studies. Chronic renal failure is a continuous psychological process for patients and their families in order to accept their new image and to be adjusted to the new condition of hemodialysis [6]. Studies have explored the experience of anxiety and depression in patients with ESKD in the context of renal failure [7]. Sein et al. [8] studied emotional distress and adjustment in patients with end-stage renal disease. They found that many patients felt it was inappropriate to disclose their emotional problems to nephrology staff because they believed staff lacked the time to deal with such issues or believed staff lacked the necessary skills to provide solutions. In addition, Patients receiving hemodialysis also have psychological symptoms, poor adaptability to the disease, fewer steps to seek treatment, and lower quality of life [4]. As a result, patients may experience many emotional and psychological problems [9]. It is also important to help patients build adjustment and coping mechanisms [8]. Consequently, chronic kidney disease poses a grave threat to public health, demanding heightened attention from healthcare providers towards these vulnerable patients.

Culture plays a significant role in influencing the health behavior, disease cognition, response, and medical-seeking behavior of ESRD patients [10]. Taiwan is a multicultural society comprising diverse Han subgroups, as well as indigenous Malayo-Polynesian peoples and immigrants from all over the world [11]. As the number of HD patients continues to rise, it becomes essential for HD nurses to possess cultural knowledge to cater effectively to the diverse healthcare needs of patients from various cultural backgrounds [12]. In this increasingly diverse society, the importance of nurses’ cultural competence cannot be overstated. It is crucial to address the health needs of HD patients from different cultural backgrounds and reduce health disparities [13]. Studies conducting systematic literature reviews have pointed out the significance of culturally competent healthcare workers in improving individual health outcomes. Patients receiving culturally sensitive nursing care have reported higher satisfaction, while culturally competent clinical nurses have contributed to improved care quality and better health outcomes [14]. Many studies have revealed that the enhancement of cultural competence requires training and continuous, time-consuming efforts. It cannot be achieved through a few years of school education alone. As such, cultural competence should be incorporated into the lifelong learning courses offered by healthcare institutions [15,16].

Previous studies have reported the effectiveness of using “simulation” as an educational strategy for nursing professionals, enabling learners to engage actively in a safe environment and enhancing various aspects of healthcare workers’ development, including cognition, skills, self-efficacy, self-confidence, and communication [17]. This approach emphasizes acquiring meaningful knowledge within the context of the situation, encouraging learners to observe, find clues, utilize resources, and propose solutions actively, thereby facilitating knowledge transfer [18]. Simulation teaching involves an interactive learning process that encompasses both comprehension and experiential learning, with clear objectives, dynamic and engaging clinical scenarios, and ultimately relates the experience to clinical realities through debriefing [11]. Debriefing, on the other hand, generally involves a guided and detailed discussion among a group of individuals who have collectively experienced an event [19,20]. In the context of simulation teaching, simulation debriefing refers to a two-way interactive discussion after the simulation. Participants engage in self-reflection and an analysis of their behavior and performance [21]. Feedback plays a pivotal role in the simulation debriefing process, often used for debriefing participants with limited experience, as it helps enhance their dialogue skills [22]. Consequently, integrating effective feedback into the debriefing process is essential and indispensable for current clinical skills teaching [23]. The Gather–Analyze–Summarize (GAS) method is one of many three-step debriefing structures identified by Sawyer et al. [22]. The GAS debriefing process can be divided into three distinct stages, each offering structural and supportive features. During the first stage (Gather), participants have the opportunity to share their thoughts on the simulated situation and engage in an exchange in ideas to collectively build a shared mental model of the situation. In the second phase (Analyze), learners can partake in a learner-centered discussion of the simulation, guided by tailored questions aligned with specific learning objectives, enabling them to understand the areas of strength and areas for improvement in their performance. Finally, in the Summarize stage, participants review the entire teaching plan and analyze the process with focused attention before the conclusion of the simulation teaching. This practice and analysis process ensures that learners genuinely comprehend the learning objectives and priorities of the course [23,24]. Several studies have indicated the importance of the cultural competence of HD nurses in caring for dialysis patients [13,25]. Krueger [25] conducted qualitative interviews with 23 HD nurses to understand the experience of patients receiving HD. The results of the study showed that HD nurses could comprehend the cultural background of HD patients through various means; however, there remains a lack of methods for culture training, including issues related to cultural challenges, beliefs about treatment and disease, and fears related to HD treatment. Furthermore, Mahabeer [13] conducted a survey on the cultural competence of HD nurses. The results showed that some HD nurses were not cognizant that factors such as the dietary and cultural beliefs of HD patients could interfere with treatment. For example, HD patients may use herbs or alternative medicine as part of their cultural beliefs; however, these treatments may have interactions with prescribed treatment. A review of the relevant literature revealed a relative scarcity of studies on the cultural competence of HD nurses in Taiwan. Given the specificity and individuality of culture, nursing professionals must address the cultural diversity present in the HD patient population, necessitating nursing plans that consider multiple criteria. Therefore, the present study aimed to integrate the GAS method of debriefing into situated simulation education to explore the effect of cultivating cultural competence among HD nurses.

## 2. Materials and Methods

### 2.1. Design and Sample

This study employed a one-group, quasi-experimental design. Using convenience sampling, data were collected from November 2022 to January 2023 at an HD center with 386 HD patients in northern Taiwan. There were 45 HD nurses at the selected hospital. The participants of this study were licensed nurses who had received a formal nursing education, and had been working in their job for at least 1 year. All the participants had the same cultural background. Nurses with depression or malignant disease may not only suffer, but their illness may affect their colleagues, as well as the quality of care they provide [26,27]. Therefore, these nurses were excluded from this study. The sample size was estimated using the G*power statistical software package, version 3.1.2, with the power set at 0.8, a significance level of 0.05, and an effect size of 0.40. The sample size required was calculated to be 36. Accounting for the potential attrition rate, the final estimated sample size was set at 40.

### 2.2. Measurements

#### 2.2.1. Participants’ Characteristics

The participants’ characteristics were derived from the study by Liang et al. [28], which included age, marital status, educational attainment, nursing seniority, department, experience in caring for foreigners, and experience of attending cultural courses.

#### 2.2.2. Nursing Cultural Competence Scale

The Nursing Cultural Competence Scale (NCCS) is a self-reported measurement tool with 19 questions categorized under the following 4 dimensions [29]: cultural awareness ability, cultural action ability, cultural resources application ability, and self-learning cultural ability. The responses to the NCCS are rated using a 5-point Likert scale: “always” (five points), “often” (four points), “neutral” (three points), “neutral” (two points) and “rarely” (one point). The higher the score a subject received, the greater the cultural competence the subject exhibited. The overall Cronbach’s alpha was 0.92, with subscale values ranging from 0.90 to 0.83 in the present study.

### 2.3. Intervention Course

The intervention strategy in this study was based on the framework proposed by Shore [30], where the curricular model of cross-cultural sensitivity was applied to design an intercultural curriculum framework for physical therapy students. The total intervention duration was six hours, consisting of three hours of basic concept courses on cultural competence and three hours of situated simulation teaching.

#### 2.3.1. Basic Concepts of Cultural Competence

The course content was based on the cultural competence framework for clinical nurses in Taiwan, developed by Lin et al. [29], which consisted of the following areas: (1) Self-learning cultural ability: enriching one’s understanding through reading books, watching medical videos, and attending cultural or language education courses; (2) Cultural resources application ability: the ability to seek various resources to meet the cultural needs of patients with diverse cultural backgrounds; (3) Cultural action ability: the proficiency to provide nursing activities that cater to the needs of individuals with different cultural backgrounds, after evaluating their cultural backgrounds; and (4) Cultural awareness ability: the capacity to recognize that one’s own cultural views may differ from those of others, such as being aware that patients or caregivers may decline treatment due to folklore taboos, and that death is a forbidden topic [29]. Based on the cultural competency framework described above, a 3 h curriculum consisting of three units was developed for this study according to a review of the nursing workforce literature. Unit 1, titled “Your Feelings, I Understand”, aimed to elucidate how to coexist with and respect different cultures. Unit 2, titled “Kidney Health for Life”, focused on recognizing and understanding cultural concepts related to HD, fostering respect for such cultures. Unit 3, titled “Implementing Multicultural Care in Hemodialysis”, delved into the practical aspects of providing multicultural care for HD patients.

#### 2.3.2. Integrating the GAS Method of Debriefing into the Cultural Competency Situated Simulation Program

This study examined the psychological adjustment process commonly observed in HD patients during clinical practice through the portrayals of HD patients faced multicultural scenarios by a standard patient (SP), with three teaching scenarios, each with an appropriate and an inappropriate version. Scenario 1 aimed to assist patients in managing the psychological impact of HD, particularly in dealing with disability by recognizing and accepting patients’ emotions. The objective was to guide patients towards emotional composure and adjustment of their condition, moving from resistance to accepting the disease. In Scenario 2, empathy was expressed towards the patients’ multicultural treatment choices to ensure that they had a clear understanding of the options related to HD treatment. The focus of Scenario 3 was on encouraging patients to express their emotions openly. Through guidance, patients were prompted to articulate their expectations and attitudes towards disease care, facilitating an open expression of their thoughts on HD (Box 1).

Box 1 **Simulation scenarios of the study.**

◆Scenario 1

   Mr. Wang presented with full-body edema and shortness of breath, prompting the doctor to recommend hemodialysis. However, Mr. Wang strongly resisted the idea of undergoing dialysis, as he felt that throughout his illness, Western medicine had offered no cure, only management of the condition. Desperate to avoid dialysis, he attempted various remedies, but his toxin levels continued to rise, leaving him with no choice but to undergo kidney dialysis. He could not understand why the remedies that worked for others had no effect on him. He expressed concern for his wife, who now had to care for him. The loss of kidney function had already taken a toll on his self-esteem, and his inability to work added to the burden on his family.

◆Scenario 2

   Mr. Wang recalled that when he was first diagnosed, people suggested various folk remedies to him. Eager to find a solution, he tried these remedies one after the other, but unfortunately, none proved effective in the long run. While some provided temporary relief, his discomfort returned, and the toxins in his body continued to rise, leading to alarming episodes of unconsciousness. Feeling desperate, Mr. Wang shared, “Living with this disease feels like a constant struggle between life and death. I try to delay hemodialysis for as long as possible, but it seems inevitable. If I couldn’t delay it any further, I might as well accept my fate and die”. When the attending physician visited his ward, the doctor, being a specialist in the field, suggested giving dialysis a try for half an hour. To Mr. Wang’s surprise, the procedure turned out to be less daunting than he anticipated. He described that it felt similar to an intra-vein injection, with just a prick from the needle, causing discomfort for only a very short period of time.

◆Scenario 3

   Mr. Wang closed his eyes, repeatedly expressing his fear that his condition might not be curable, and he would have to undergo kidney dialysis, which he perceived as the end of his life. Over a period, he faithfully received hemodialysis three times a week as scheduled. He told the nurse, “Initially, I believed that dialysis would burden my family, especially my wife. However, with time, I realized that it wasn’t as terrible as I had imagined. The most significant thing is that I feel more comfortable after each session. The drawback is having to repeat it every few days, and the needle pricks are painful. I can’t help but feel pity for myself, and it’s inconvenient to go anywhere since I have to consider the availability of hospitals with dialysis facilities”. Gradually, Mr. Wang found the pain bearable, and despite his illness, he felt that he could still function quite well and found value in being alive. He even considered himself fortunate in some ways. His main message was that unexpected accidents can take away a person’s life and the chance to express themselves, but he, like others undergoing hemodialysis, had the opportunity to survive and continue living through hemodialysis, which was much better than for those who experienced sudden unexpected accidents.

Situated simulation teaching began with the introduction of learning objectives and an explanation of the group teaching method. Subsequently, participants observed a psychological adjustment process commonly experienced by HD patients, portrayed by the SP. HD nurses then engaged in effective nurse–patient communication regarding the first situated simulation (inappropriate version) for approximately 10 min, followed by a 10 min debriefing. Next, participants viewed the SP’s portrayal of the psychological adjustment process of clinical HD patients for a second time (proper version) for 20 min. This was followed by a 10 min discussion and a subsequent 10 min debriefing session. In order to enhance the learning effect and time constraints, only the appropriate version of the situation was discussed. The GAS debriefing method was integrated into the three clinical simulation scenarios involving the psychological adjustment process of clinical HD patients portrayed by the SP. In the first stage (Gather), participants were prompted to review the scenario and encouraged to share their thoughts, allowing for an assessment of potential cognitive gaps between the learners and instructors. Questions like “How did everyone perform just now?” were used to facilitate this process. This stage constituted 25% of the briefing time. The second stage (Analyze) involved assessing the participants’ performance, allowing them to recognize areas of strength and areas for improvement. Guided by appropriate statements such as “Yes, what everyone is discussing is very important, but I would like to ask you to focus on…”, this stage accounted for 50% of the briefing time. In the third stage (Summarize), the entire teaching plan was reviewed and analyzed to ensure a comprehensive understanding of the course objectives and priorities. Participants were asked questions, such as “Please identify two aspects that you believe could be done differently next time”. This stage occupied 25% of the briefing time. The cultural competency situated simulation teaching lasted for three hours.

### 2.4. Data Collection

**Figure 1:**
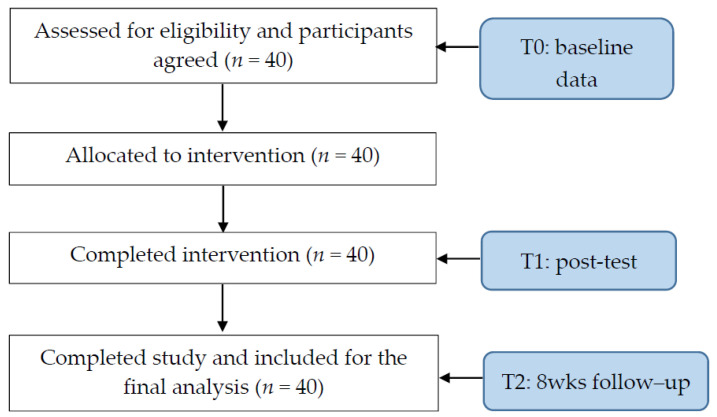
Flow diagram of the study.

Participants were recruited after the researchers had obtained approval from the institutional review board of the institution with which they were affiliated, explained the study to the participants, and obtained written consent from the latter. Prior to the intervention, the participants received a pre-test, the answers to which served as the baseline (T0). A post-test (T1) was conducted immediately after the intervention ended, which revealed the immediate effect of the intervention. In addition, follow-up data collection was conducted eight weeks after the intervention (T2) to evaluate the delayed effect. The subjects participated in the intervention program, which consisted of a 3 h course covering the basic cultural concepts and a 3 h situated simulation teaching session on the clinical practice of cultural competency. Figure 1 shows the flow diagram of the study.

### 2.5. Statistical Analysis

SPSS Statistics 22.0 (IBM Inc., Armonk, NY, USA) for Windows was used for data analysis. The participants’ demographics were analyzed using descriptive analysis. The results are presented as percentages, and include mean values and standard deviations. Generalized estimating equations (GEEs) were employed to estimate the intervention effect. The baseline scores were divided into low-score and high-score groups using the median score for subgroup analysis. The low-score group was used as the reference group to examine the interaction between group and time further. The level of statistical significance was set at *p* < 0.05.

### 2.6. Ethical Considerations

This study was approved by the appropriate institutional review board (Cheng Hsin General Hospital, Taiwan). Approval for this study was obtained on 16 March 2022 with letter number CHGHIRB-(887)110A-33. No individuals were identified in any reports or publications resulting from this study. The participants were given a clear explanation of the study objective by the investigator, and their oral and written consent was obtained. The investigator also provided detailed information about the participant’s rights and obligations with respect to the research process, with a focus on ensuring the anonymity of data collection. Participants were assured that their completed questionnaire data would be kept confidential and would solely be used for academic research purposes. Furthermore, participants were informed of their right to withdraw from the study at any point without incurring any fees if they felt uncomfortable during the process.

## 3. Results

### 3.1. Demographic Variables of Participants

The study participants had a mean age of 43.27 (±6.60) years, and their overall mean nursing experience was 20.62 (±7.79) years, with an average of 12.68 (±9.08) years of service specifically in the HD unit. Among the participants, 52.5% were unmarried, 67.5% had a university education, and 70% held N1 or N2 professional ranks. Additionally, 62.5% of them had experience in caring for foreigners, and 35.0% had participated in cultural courses (Table 1).

### 3.2. Distribution of the Scores Obtained from the Three Tests on the NCCS Total and Subscale Scores

The mean nursing cultural competence scores for the baseline, post-test, and 8-week follow-up tests were 2.68, 2.94, and 2.67, respectively, indicating a significant decrease in the cultural competence delayed effects after the intervention. Overall, the results showed that the scores of the measurements the three different times revealed small fluctuations up and down regardless of the cultural awareness ability, cultural action ability, cultural resources application ability, and self-learning cultural ability in the four subscales (Table 2).

### 3.3. The Effects of the Educational Intervention on the Cultural Competence of the Participants

To understand the impact of the educational intervention and its various dimensions on the overall cultural competence of the study participants, the GEE model was employed for analysis. The exchangeable correlation matrix was used to control the effect of time, and robust standard error was utilized to calculate the significance [31]. To analyze the baseline scores collected before the Intervention, the participants were categorized into low-score and high-score groups based on the median score, followed by subgroup analysis. The low-score group was used as the reference group to examine the interaction between group and time further.

According to the subgroup analysis, the results revealed that the NCCS total and subscale scores of the high-score group were higher than those of the low-score group, with the difference reaching statistical significance (*p* < 0.001). The improvement scores of the total cultural competence, cultural awareness ability, cultural resource application ability, and self-learning cultural ability of the high-score group were found to be 0.38, 0.99, 0.65, and 0.66, respectively, lower than those of the low-score group (*p* = 0.03, <0.001, =0.02, =0.001, respectively) at post-test, which indicated a significant improvement in the total cultural competence and subscale score of the low-score group immediately after the intervention. In terms of cultural awareness ability and cultural resources application ability, the improvement scores of the high-score group were 0.95 and 0.55, respectively, lower than those of the low-score group (*p* = 0.001, = 0.04, respectively) at the follow-up test (Table 3).

## 4. Discussion

The objective of this study was to develop a tailored situated simulation training program for HD nurses, incorporating the three-stage debriefing approach of the GAS method. Employing a one-group intervention pilot study design, the effectiveness of the training program was evaluated by assessing participants’ cultural competence before, immediately after, and eight weeks post-intervention. Based on the baseline scores obtained before the intervention, the participants were divided into low-score and high-score groups using the median score, and further subgroup analysis was performed. The findings indicated a significant main effect of time on the total cultural competence, cultural awareness ability, cultural resources application ability, and self-learning cultural ability scores, with scores showing an increase over time. The findings of this study aligned with those of previous research, suggesting that increasing the training time can lead to a higher degree of measured behavior change, further underscoring the effectiveness of the intervention [32,33]. The educational intervention hours in this study totaled six hours, comprising three hours of education on the basic concepts of cultural competence and three hours of situated simulation training. Cerezoa et al. [34] revealed that the duration of intervention in cultural competence courses can vary widely, ranging from three hours to several days or even weeks. However, Chen et al. [33] found no significant difference in cultural competence between the control group and the experimental group after participants completed cultural courses, followed by 10 h of on-the-job training. Conversely, Chang et al. [35] and Stiles et al. [36] demonstrated that participants with more than four weeks of long-term practical experience exhibited a notably positive impact on cultural competence. As different studies have yielded varying outcomes regarding the number of intervention hours and their effects on cultural competencies, careful consideration in future follow-up studies is warranted.

This study further investigated the immediate and delayed effects of situated simulation education on participants in the low-score group, who had lower baseline scores at the beginning. Overall, the results indicated that participants in the low-score group with lower baseline scores showed greater improvement over time after the intervention in the dimensions of total cultural competence, cultural awareness ability, cultural resources application ability, and self-learning cultural ability, suggesting a more pronounced immediate effect on these participants. Furthermore, regarding the delayed effect of the intervention, the low-score group also exhibited more noticeable improvements in cultural awareness ability and cultural resources application ability. These findings aligned with those of a survey conducted by Cicolini et al. [37] with Italian nurses, which revealed that the nurses had acquired a certain level of cultural awareness and sensitivity. The current study utilized a one-group experimental design, and the results demonstrated immediate and delayed effects on the NCCS total and subscale scores of the low-score group. These findings were also consistent with those of Horvat et al. [38], who pointed out the importance of incorporating multiple teaching strategies in cultural competence courses. In this study, the intervention strategy involved designing teaching scenarios based on the psychological adjustment process commonly observed in HD patients in clinical practice. SPs were used to portray multicultural scenarios of HD patients for situated simulation teaching of cultural competence, followed by the GAS method of debriefing. The GAS method debriefing included clear time allocation, goals, priorities, and suggested example sentences, providing high-quality debriefing within a systematic framework [16]. Moreover, the integration of “reflection” as an important activity design [15] further enhanced the intervention strategy. Haugan et al. [39] emphasized the significance of focusing on reflection and feedback in cultural competence education. The reflection process was guided not only by relevant theoretical knowledge and feedback but also by theoretical topics integrated into the practice of clinical experience through reflection. Thus, it is likely that the intervention strategy played a crucial role in improving the performance of the low-score group in this study.

It is noteworthy that the results of this study indicated that there were no significant main effects of time or interaction in the cultural action ability dimension. As defined by Lin et al. [18], cultural action ability refers to the extent to which nursing staff can address misunderstandings arising from language barriers, how different cultures influence treatment policies, and the impact of various religious rituals or lifestyle habits on nursing care. The lack of effectiveness in the cultural action ability intervention may be attributed to the frequent nature of HD treatment, which occurs three times a week for most HD patients. HD nurses demonstrated their willingness to empathize with and accept patients’ behavior through observation and the seeking of further clarification to understand the significance of dialysis for each patient. Consequently, participants’ cultural action ability seemed unaffected by the educational intervention. Further exploration and discussion of this aspect should be considered in future follow-up studies.

Furthermore, it is important to acknowledge that the use of self-administered scales in this study may have influenced the research results. As noted by Schellings et al. [40], self-administered questionnaires often rely on participants’ self-assessment and inference of their own activities. While this approach offers advantages, such as minimal disruption during the learning activity and ease of administration in large-scale testing, it may also introduce psychological biases, such as social desirability, into participants’ responses during the questionnaire administration. This could lead to inaccuracies in reporting their usual behaviors or activities after completing the task. Additionally, self-administered questionnaires may primarily measure participants’ views rather than their actual implementation strategies, making it challenging to obtain objective measurements of their attitudes. Consequently, the validity of the self-administered questionnaire data could be compromised, indirectly affecting the reliability of the study results [28]. To enhance the understanding of the participants’ learning experience and responses to the intervention measures, this study could have benefited from the inclusion of observation records of teaching activities to complement the quantitative data. While the use of self-administered scales represents a limitation in this study, the findings reveal a significant and major effect of time on the total cultural competence, cultural awareness ability, cultural resource application ability, and self-learning cultural ability dimensions. The scores showed an increase over time, indicating the value of the educational intervention content and teaching strategies in fostering cultural competence among the research participants. This study integrated the GAS method of debriefing into the cultural competency situated simulation program in guiding patients towards emotional composure. Composure is the state or feeling of being calm and in control of oneself [41]. Emotionally stable persons tolerate minor stresses and able to maintain composure under minor emotional stress [42]. Generally, increased levels of composure during interactions lead to more positive outcomes [43]. According to Austin [44], qualitative research is concerned with participants’ own experiences of a life event, and the aim is to interpret what participants have said in order to explain why they have said it. Future research should combine quantitative and qualitative methods to explore the psychological adjustment process and established, determined and/or measured emotional composure among HD patients.

The results suggested positive changes due to the pre-, post-, and follow-up interventions. However, there are several limitations to this study. First, this study was quasi-experimental, which may have limited its external validity. Randomized controlled trials could be used to measure the effectiveness of the cultural competence intervention. Second, the intervention was implemented in a local hospital’s HD center; therefore, the results cannot be generalized to other institutions. Third, this study was affected by the SARS-CoV-2 pandemic. Nurses were under great pressure, which may have led to overestimations or underestimations of the effectiveness of the intervention. Future studies should include a control group and a larger sample size. Finally, this study adopted convenience sampling, and the majority of participants were female. Hence, the results cannot be extrapolated to all HD nurses. Despite these limitations, this study added to our understanding of the cultural competency of HD nurses by providing intervention results regarding participants’ participation in cultural competency programs.

## 5. Conclusions

The results of this study demonstrated the positive effects of integrating the GAS method of debriefing into situated simulation education, enhancing the cultural competence among HD nurses. Although the intervention did not result in significant differences in cultural competence among all participants, a progressive improvement in the scores was observed, particularly in the low-score group. These participants exhibited both immediate and delayed effects following the educational intervention, as indicated by the NCCS total and subscale scores. These findings validate the clinical applicability of the situated simulation program and demonstrate professional expertise in an organized manner when facing similar clinical scenarios in the future.

## Figures and Tables

**Table 1:** Sociodemographic characteristics of participants.

Variables		*n*	%
Gender	Male	2	5.0
	Female	38	95.0
Marital status	Married	19	47.5
	Unmarried	21	52.5
Educational attainment	Diploma	13	32.5
	Bachelor’s degree	27	67.5
professional nursing level	N1	14	35.0
	N2	14	35.0
	N3	12	30.0
Experience in caring for foreigners	yes	25	62.5
	no	15	37.5
Experience of attending cultural courses	yes	14	35.0
	no	26	65.0
		mean	SD
Age (year)		43.27	6.60
Nursing seniority (year)		20.62	7.79
Nursing seniority in HD (year)		12.68	9.08

**Table 2:** Distribution of the scores obtained from the three tests on NCCS total and subscale score.

Variables	T0		T1		T2	
	Mean	SD	Mean	SD	Mean	SD
Total score ^a^	2.68	0.73	2.94	0.79	2.67	0.88
Cultural awareness ability	2.36	0.92	2.59	1.04	2.32	0.99
Cultural action ability	2.65	0.91	2.86	1.01	2.48	1.01
Cultural resource application ability	2.99	1.01	3.37	1.07	3.09	1.09
Self-learning cultural ability	2.71	0.88	2.92	0.94	2.78	1.34

^a^: Total cultural competence; NCCS: Nursing Cultural Competence Scale; T0: baseline test; T1: post-test; T2: follow up test; SD: standard deviation.

**Table 3:** The effects of intervention on NCCS total and subscale scores of participants.

Parameter	B (95% CI)	Std. Error	Wald *χ*^2^	*p*
Total score ^a^				
Intercept	2.12 (1.92, 2.3251.52)	0.10	449.29	<0.001
Group ^b^				
Low score vs. high score	1.20 (0.94, 1.46)	0.13	81.11	<0.001
Time				
T1-T0	0.36 (0.08, 00.64)	0.14	6.44	0.011
T2-T0	0.29 (0.06, 0.52)	0.11	6.25	0.012
Group × Time ^c^				
high score × T2	−0.39 (−0.80, 0.01)	0.21	3.50	0.06
high score × T1	−0.38 (−0.75, −0.02)	0.18	4.33	0.03
Cultural awareness ability				
Intercept	1.54 (1.40, 1.68)	0.07	471.35	<0.001
Group ^b^				
Low score vs. high score	1.77 (1.38, 2.17)	0.20	78.30	<0.001
Time				
T1-T0	0.55 (0.12, 0.98)	0.21	6.49	0.01
T2-T0	0.33 (0.09, 0.58)	0.12	7.27	0.007
Group × Time ^c^				
high score × T2	−0.95 (−1.49, −0.41)	0.27	12.08	0.001
high score × T1	−0.99 (−1.54, −0.43)	0.28	12.16	<0.001
Cultural action ability				
Intercept	2.16 (1.89, 2.42)	0.133	261.81	<0.001
Group ^b^				
Low score vs. high score	1.38 (1.07, 1.69)	0.16	74.90	<0.001
Time				
T1-T0	0.32 (−0.11, 0.75)	0.21	2.12	0.14
T2-T0	0.06 (−0.23, 0.36)	0.15	0.19	0.66
Group × Time ^c^				
high score × T2	−0.48 (−1.01, 0.04)	0.26	3.28	0.07
high score × T1	−0.43 (−0.95, 0.09)	0.26	2.58	0.10
Cultural resources application ability				
Intercept	2.23 (1.99, 2.48)	0.12	319.19	<0.001
Group ^b^				
Low score vs. high score	1.65 (1.31, 2.00)	0.17	88.20	<0.001
Time				
T1-T0	0.63 (0.12, 1.14)	0.25	5.97	<0.001
T2-T0	0.44 (0.05, 0.83)	0.19	4.94	0.01
Group × Time ^c^				
high score × T2	−0.55 (−1.08, −0.01)	0.27	4.05	0.04
high score × T1	−0.65 (−1.23, −0.07)	0.29	4.86	0.02
Self-learning cultural ability				
Intercept	2.28 (2.06, 2.50)	0.11	403.20	<0.001
Group ^b^				
Low score vs. high score	1.46 (1.13, 1.79)	0.16	75.01	<0.001
Time				
T1-T0	0.35 (0.02, 0.69)	0.17	4.37	0.03
T2-T0	0.69 (0.32, 1.05)	0.18	14.02	<0.001
Group × Time ^c^				
high score × T2	−0.69 (−1.42, 0.04)	0.37	3.43	0.06
high score × T1	−0.66 (−1.05, −0.26)	0.20	10.79	0.001

NCCS: Nursing Cultural Competence Scale; T0: baseline test; T1: post-test; T2: follow up test; ^a^ Total cultural competence; ^b^ Reference: low score; ^c^ Reference: low score group × baseline T0.

## Data Availability

All relevant datasets in this study are described in the manuscript.
